# Morphological Evidence for the Sensitivity of the Ear Canal of Odontocetes as shown by Immunohistochemistry and Transmission Electron Microscopy

**DOI:** 10.1038/s41598-020-61170-4

**Published:** 2020-03-06

**Authors:** Steffen De Vreese, Michel André, Bruno Cozzi, Cinzia Centelleghe, Mike van der Schaar, Sandro Mazzariol

**Affiliations:** 1 Department of Comparative Biomedicine and Food Science, University of Padova, 35020 Legnaro, Padova Italy; 2 Laboratory of Applied Bioacoustics, Technical University of Catalunya, BarcelonaTech, 08800 Vilanova i la Geltrú, Barcelona Spain

**Keywords:** Developmental biology, Evolutionary developmental biology

## Abstract

The function of the external ear canal in cetaceans is still under debate and its morphology is largely unknown. Immunohistochemical (IHC) analyses using antibodies specific for nervous tissue (anti-S100, anti-NSE, anti-NF, and anti-PGP 9.5), together with transmission electron microscopy (TEM) and various histological techniques, were carried out to investigate the peripheral nervous system of the ear canals of several species of toothed whales and terrestrial Cetartiodactyla. This study highlights the innervation of the ear canal with the presence of lamellar corpuscles over its entire course, and their absence in all studied terrestrial mammals. Each corpuscle consisted of a central axon, surrounded by lamellae of Schwann receptor cells, surrounded by a thin cellular layer, as shown by IHC and TEM. These findings indicate that the corpuscles are mechanoreceptors that resemble the inner core of Pacinian corpuscles without capsule or outer core, and were labelled as simple lamellar corpuscles. They form part of a sensory system that may represent a unique phylogenetic feature of cetaceans, and an evolutionary adaptation to life in the marine environment. Although the exact function of the ear canal is not fully clear, we provide essential knowledge and a preliminary hypothetical deviation on its function as a unique sensory organ.

## Introduction

The morphology of the external ear canal of toothed whales has received little attention in comparison to the middle and inner ear (e.g^1,2^.), and there is a debate on whether the canal still serves any function^3^. The exact pathways for sound reception are not yet fully understood but it is known that echolocation signals are received through the mandibular fat bodies, while other sounds, such as those for communication, could be received through a lateral soft tissue pathway^3–5^. However, whether the external ear canal forms a functional part of this or any other process is still a conundrum, and even basic knowledge on its morphology is largely incomplete. Very few papers describe the morphology of the ear canal in cetaceans and even fewer mention the presence of lamellar corpuscles, likely mechanoreceptors^6,7^. Similar corpuscles have been mentioned in other tissues of cetaceans including the skin of the trunk, flippers, and fluke^8^, lips and eyelids^9^, inside the nasal sac system^10–12^, and associated with vibrissal crypts^13^, but their function is not clear. Moreover, there is a lack of information on the fine-scale morphology of these sensory nerve formations (SNF’s) and their role in the external ear canal.

To achieve a better understanding of the functionality of the ear canal in dolphins, we used several histological staining techniques, together with immunohistochemical labelling with four different antibodies specific for nervous tissue antigens. We also applied TEM to study the fine-scale morphology of the lamellar corpuscles, and did a preliminary quantitative study on their distribution along the ear canal. We compared the peripheral nervous system of the ear canals of several odontocete species (i.e. striped dolphin, bottlenose dolphin, common dolphin, long-finned pilot whale and Cuvier’s beaked whale) with those of several terrestrial Cetartiodactyla (i.e. cow, roe deer and northern giraffe).

## Results

### Striped dolphin

**Figure 1:**
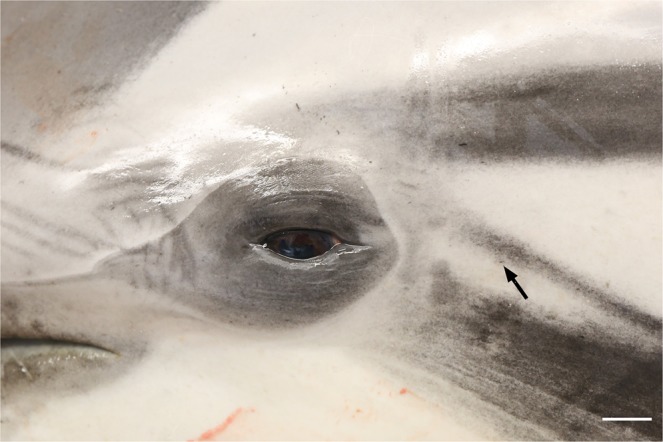
Macroscopic view of the left lateral side of a young striped dolphin head showing the position of the inconspicuous aperture of the external ear canal situated a few centimetres caudal to the eye (arrow). (Scale bar 1 cm).

**Figure 2:**
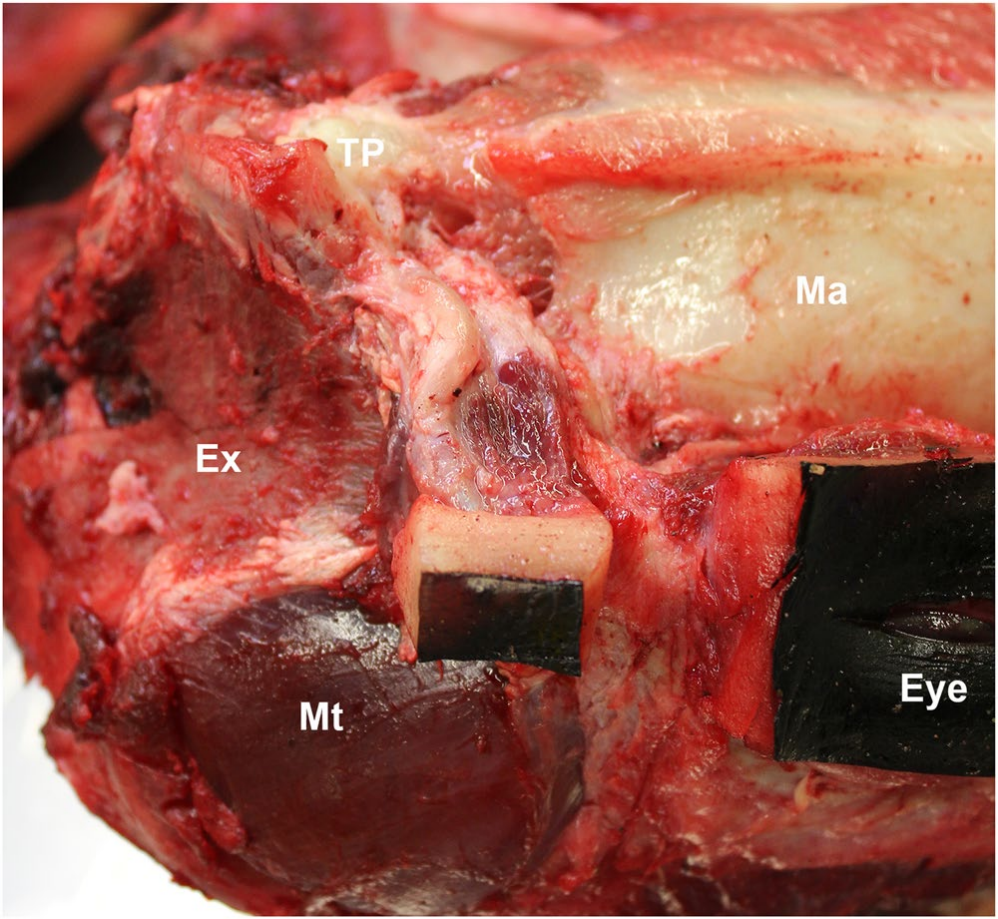
Macroscopic image in left lateral view of the course of the external ear canal of striped dolphin. The head is positioned upside-down. Note the spiralling course of the canal in caudoventral direction, from the skin, through the blubber, soft tissues and associated musculature, as it reaches the TP complex medially. The canal enters the parotic cavity in the caudodorsal margin of the trench created by the caudal ramus of the mandible rostrally, the retrotympanic and retroarticular (postglenoid) process of the squamosal dorsally, and the lateral margin of the exoccipital bone (Ex) caudally. Mt: temporal muscle.

**Figure 3:**
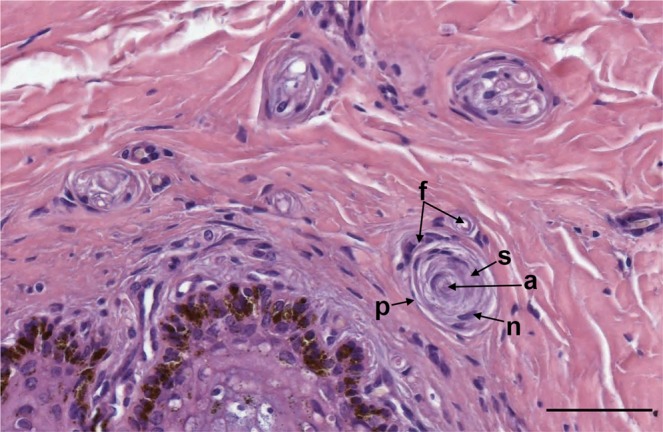
HE staining of simple lamellar corpuscles and small nerve bundles in the vicinity of the external ear canal in striped dolphin. a: central axon; n: Schwann cell nuclei; s: Schwann cell cytoplasm and cell membrane; p: peripheral layer; f: nerve fibres (or small vascular tissue) associated with the peripheral layer of the corpuscle. (Scale bar 50 µm).

**Figure 4:**
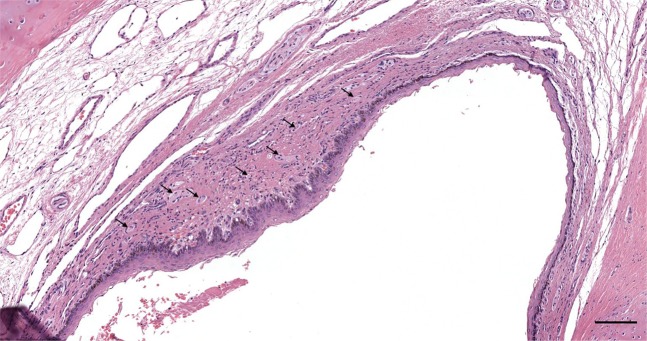
HE stained section through the cartilaginous portion of the ear canal of striped dolphin. Note the local bulge of richly innervated tissue with heightened epithelium and papillae. The arrows indicate the location of several nervous structures. (Scale bar 100 µm).

**Figure 5:**
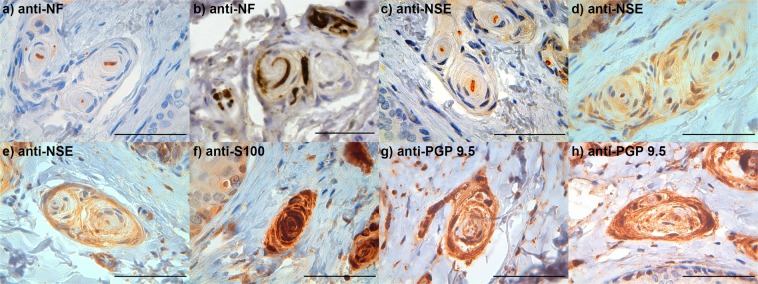
Immunohistochemical stained microscopic images of lamellar corpuscles in striped dolphin stained with (**a,b**) anti-NF, in (**b**) note the whirling course of the central axon within a composite corpuscle and several axons associated with the peripheral layer, (**c**–**e**) anti-NSE, note the immunoreactivity of the central axon and inconsistent staining of the peripheral layer, (**f**) anti-S-100, (**g,h**) anti-PGP 9.5. (Scale bars 50 µm).

**Figure 6:**
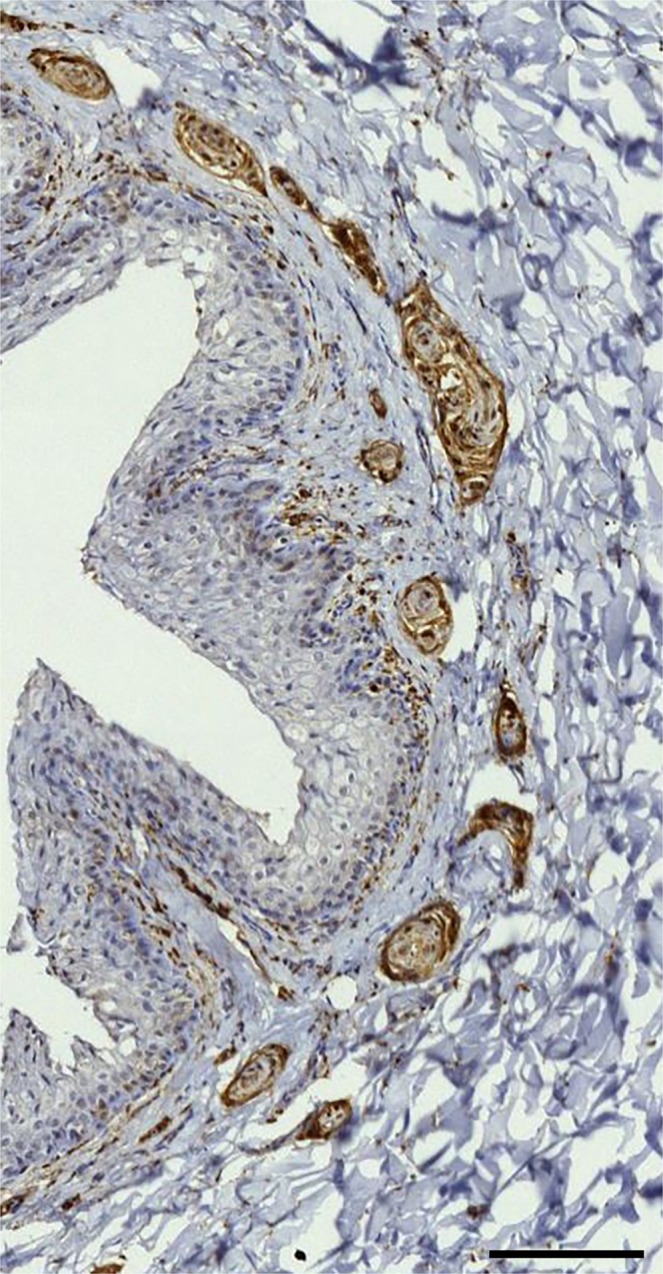
IHC staining with anti-PGP 9.5 (1:500, blocking diluent, melanin bleaching). There are single and composite lamellar corpuscles, small nerves, and the dense presence of immunoreactive spots beneath the epithelium, and also free nerve endings entering the epithelium (arrows). (Scale bar 100 µm).

Like all cetaceans, the striped dolphin lacked an external pinna. The external ear opening was visible as a small indentation of the skin situated 4–5 cm ventrocaudal to the lateral commissure of the eye at an angle of about 25–30° to the horizontal (Fig. 1). The ear canal itself ran a spiralling course in ventromedial direction through the skin, blubber, and adipose-connective tissues, and reached the tympano-periotic complex (TP-complex) over a distance of 4–5 centimetres (Fig. 2). Initial identification of nervous structures was done by standard haematoxylin-eosin staining (Fig. 3). We found lamellar corpuscles in the subepithelial tissue of the external ear canal in all sections, from superficial to the deep, of all animals. In superficial sections, the corpuscles were situated all around the meatus, while in the cartilaginous portion of the canal, corpuscles were concentrated in a tissue enlargement that bulged into the canal lumen (Fig. 4). The corpuscles were elongated with occasional convolutions and with a general course parallel to the ear canal. The diameter of the corpuscles ranged from 16 to 202 µm (geometric means, computed over the semi-minor and semi-major axes of a corpuscle, ranged from 20 to 133 µm), measured in 160 corpuscles in ten equally spaced cross-sections over the course of the canal. The larger the diameter of the corpuscle, the more lamellae it contained. Most corpuscles were singular, i.e. with a single core (axon + lamellae), but there were also composite corpuscles with multiple cores, mostly two or three, and combinations of corpuscles and nerve fascicles embedded within the same perineurium. The supplying myelinated nerve fibre lost its myelin sheath on entering the corpuscle. Each corpuscle consisted of a central axon showing immunoreactivity (IR) for anti-neurofilament protein (anti-NF), anti-neuron specific enolase (anti-NSE), and anti-protein gene product 9.5 (anti-PGP 9.5) (Fig. 5). In all sections (consecutive transverse or oblique, no perfectly longitudinal section was obtained), the terminal axon followed a straight course throughout the corpuscle, while in a single section we noted a whirling at the terminal end (Fig. 5b). Surrounding the axon, there were concentric layers (lamellae) of cells which nucleus and cytoplasm stained positive for anti-S-100 although in two gradients: the central lamellae stained more intensely positive than the peripheral ones (Fig. 5f). In contrast, anti-PGP 9.5 stained the peripheral lamellar layers surrounding a less intense positive zone in the centre (Figs. 5g,h and 6). There was IR for anti-PGP 9.5 of both cytoplasm and nuclei of the peripheral layers, but only in about half of the nuclei in the central layers (with dilution 1:500, not with higher dilutions). As such, both anti-PGP 9.5 and anti-S100 stained all lamellae but with a different zonal distribution (Fig. 5), indicating that the lamellae consisted of at least two zones with a different structural composition. The peripheral layer was thin and cellular with sparse nuclei, similar to the perineurium of small nerves. Besides the positivity for anti-PGP 9.5, this layer also showed IR for anti-NSE, staining the cytoplasm in a similar manner, although inconsistent and less intense than the staining of the axons (Fig. 5d,e). In many corpuscles, there was a non-staining space between the lamellae and the peripheral layer. Also, we often noted the presence of accessory axons, most clearly identifiable with anti-NF, but also with anti-NSE and anti-PGP 9.5, and which were situated central to the peripheral layer (Fig. 5b). Occasionally, there were also small vascular structures on the inside of the peripheral layer.

**Figure 7:**
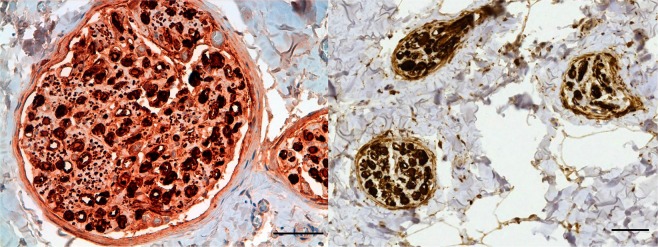
Detail image of a nerve fascicle in the subepithelial tissue in a striped dolphin stained with anti-NF (**a**) and anti-S100 (**b**). (Scale bars 50 µm).

Nerve bundles stained in a similar fashion as the lamellar corpuscles. They comprised one or more axons and associated Schwann cells embedded in an endoneurium and surrounded by a perineurium. Axons were positive for anti-NF (Fig. 7), anti-PGP 9.5 and anti-NSE. The Schwann cell nuclei and cytoplasm stained positive for anti-S100 (Fig. 7), and anti-PGP 9.5, and the perineurium for anti-PGP 9.5, anti-NSE, and anti-S-100 protein. The endoneurium did not show positivity for any antibody. The major difference in immunohistochemical labelling of corpuscles and nerves was that the perineurium was labelled intensely by anti-S-100 while the peripheral layer of the lamellar corpuscles showed more IR to anti-PGP 9.5, and while both showed IR to anti-NSE (at least in the freshest samples).

**Figure 8:**
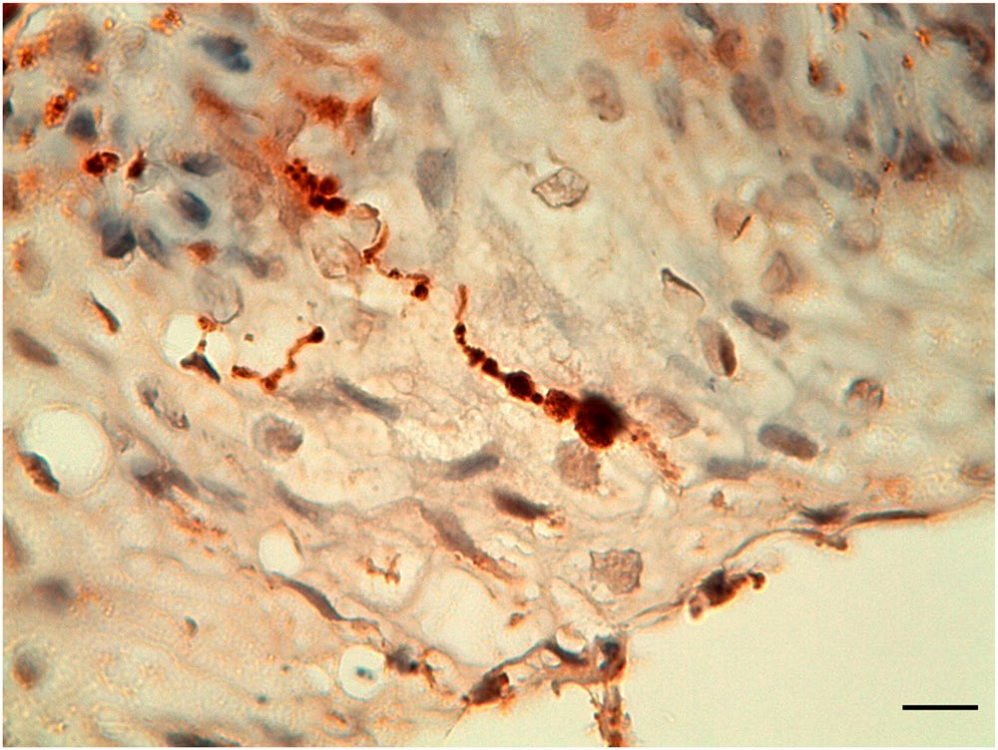
Detail image of an intraepithelial nerve fibre running from the germinal layer to the luminal surface of the ear canal of a striped dolphin, stained with PGP 9.5 (1:500, no block, melanin bleaching). (Scale bar 10 µm).

The presence of intrapapillary myelinated nerve endings (IMEs) was shown by a positive labelling with the anti-NF, anti-PGP 9.5 and anti-S-100 antibodies. All nerve fibres in the subepithelial tissue stained positive for both anti-PGP 9.5 and anti-S-100. There were positively stained singularities in the connective tissue immediately beneath the basement membrane for all antibodies except anti-NF, and there were also intraepithelial ‘free’ nerve endings as shown by anti-PGP 9.5 (Fig. 8).

**Figure 9:**
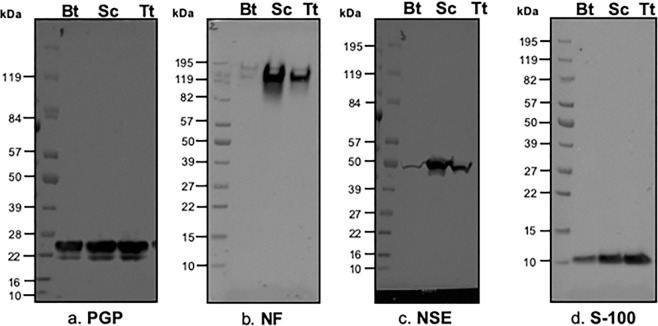
Western blot analyses. (**a**) Polyclonal rabbit anti-bovine PGP antibody (code Z5116, Dako) at a dilution of 1:500; (**b**) Monoclonal mouse anti-human NF (Clone 2F11) antibody (code M0762, Dako) at a dilution of 1:500; (**c**) Monoclonal mouse anti-human NSE antibody (Clone BBS/NC/VI-H14) (code M0873, Dako) at a dilution of 1:500; (**d**) Polyclonal rabbit anti-bovine S-100 (code Z0311, Dako) at a dilution of 1:1000. *Bt: Bos taurus, Sc: Stenella coeruleoalba, Tt: Tursiops truncatus; kDa: kilodalton.

Western blot analysis performed on the protein extracts from bottlenose dolphin, striped dolphin and bovine tissues recognized all the antigens looked for. The expression of the PGP9.5 antigen was visible at around 25 kDa (Fig. 9a) and of the NF antigen was evident at 150–160 kDa (Fig. 9b). Anti-NSE and anti-S-100 antibody reacted to the antigens showing a signal corresponding to proteins with a molecular weight of 46 kDa (Fig. 9c) and around 10 kDa (Fig. 9d), respectively.

**Figure 10:**
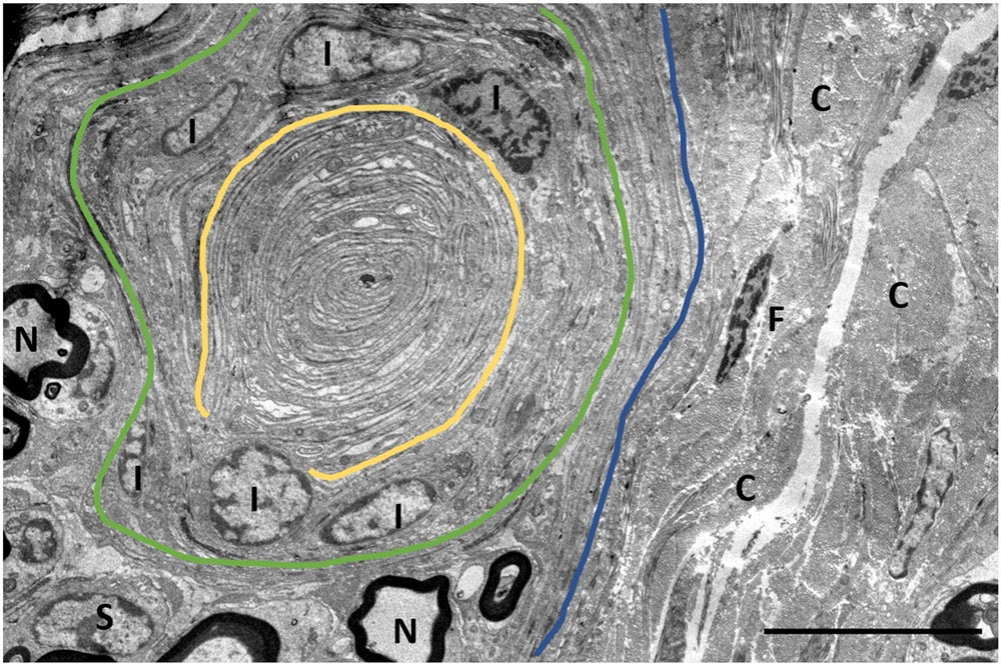
TEM Image: Overview of a lamellar corpuscles and nerve fibres (N) within the same perineurium (blue dashed line), and surrounded by collagenous connective tissue (C). Following the principles of Chouchkov (1973), the inner core with hemilamellar structure is delineated by the yellow dashed, while the capsule of the corpuscle, delineated by the green dashed line, contains the nuclei of the inner core cells (I). F: fibrocyte nucleus; S: Schwann cell nucleus. (Scale bar 10 µm).

**Figure 11:**
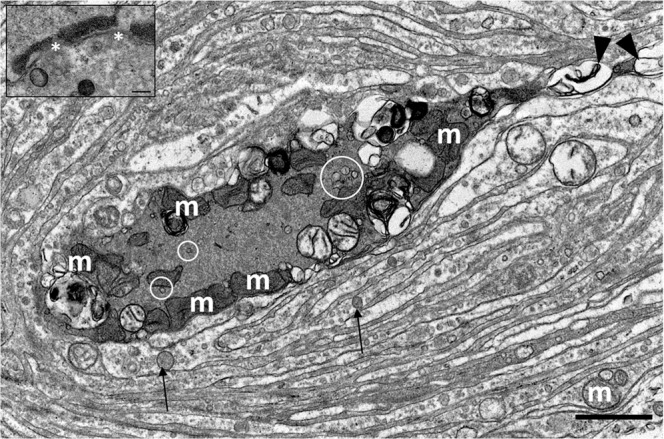
TEM image of the axon terminal (**A**) with mitochondria (m) along the rim of the neurite, and indications of microtubules (circles). The axonal spine contains large vesicular structures (arrowhead) and surrounding lamellae contain mitochondria, abundant pinocytotic vesicles, and larger vesicles (arrows). (Scale bar 2 µm). The insert shows a close-up image of two adjacent inner core lamellae with desmosome-like cell-junctions (asterisks). (Scale bar 0.1 µm).

**Figure 12:**
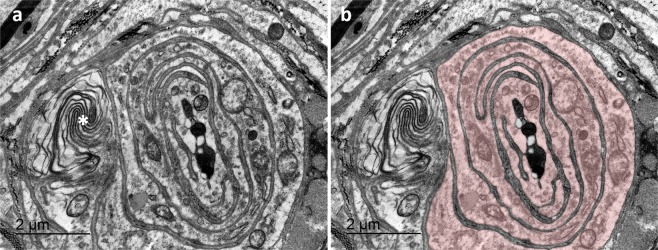
TEM image of a transverse section through the ultraterminal region of a simple lamellar corpuscle. Note that there is no bilateral symmetry, no radial clefts, and the axon is surrounded by several broad, continuous circular lamellae of a single core cell (see right image where the cytoplasm is stained). There is smooth endoplasmic reticulum (asterisk) situated in the lamellar cytoplasm. This section clearly resembles the genital end-bulb in the rat penis as described by Munger (1971^58^, Fig. 9b).

**Figure 13:**
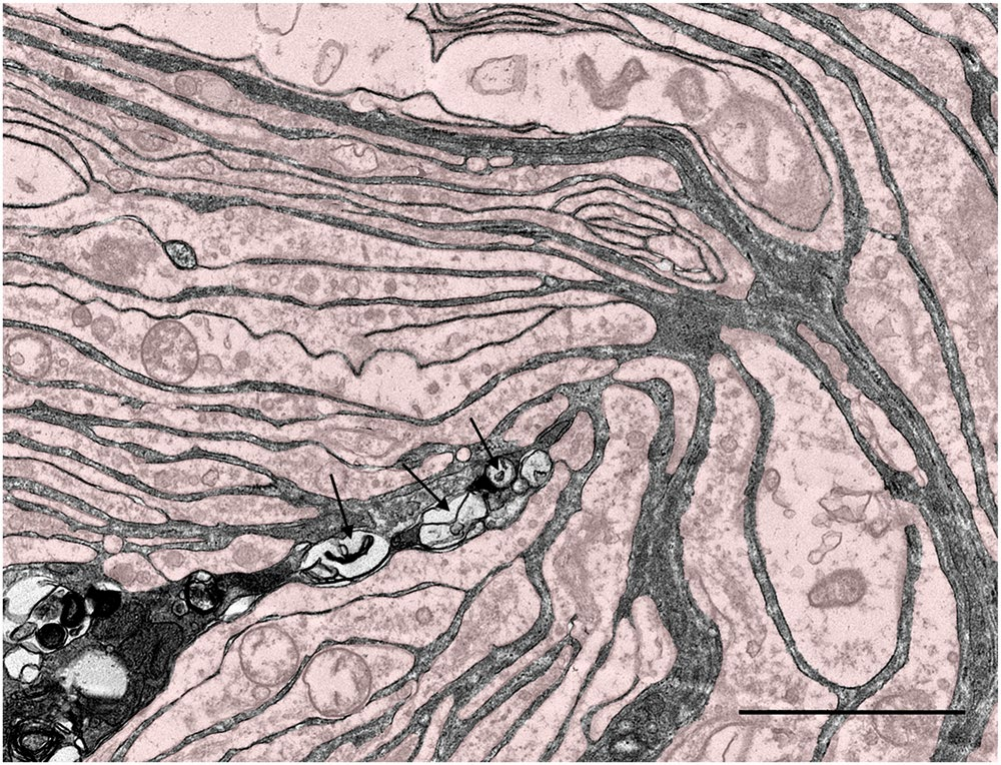
TEM image of the axonal spine protruding into a radial cleft with the lamellar cytoplasm stained slightly reddish. The spine contains clear and seemingly enlarged vesicles (arrows). (Scale bar 2 µm).

The TEM images showed that, from central to peripheral, the corpuscles were composed of an axonal (central) sensory terminal, surrounded by a lamellar core (Fig. 10). In transverse sections through the body of the corpuscles, the central axon was spindle-shaped with two opposite poles (lateral spines) (Fig. 11), while it turned oval in shape in the ultraterminal region (Fig. 12). The axon terminal contained abundant mitochondria, and vesicular-like structures of varying sizes, from large structures that often contained a nucleolus-like structure, to smaller electron-dense vesicles with a double-layered membrane, resembling synaptic vesicles, to minute, single-layered vesicles in various concentrations (Fig. 11). Interestingly, we could not identify any axoplasm with microtubules or neurofilaments with certainty, as most terminals were either almost completely filled with mitochondria or displayed a homogenous electron-dense centre with vesicular structures and mitochondria along the rim. The terminal showed bilateral axonal spines projecting into the radial clefts formed by the coming together of the bilateral hemilamellae of the inner core cells (Fig. 13). These spines contained clear vesicles with a seemingly membranous infolding and a nucleolus-like structure (Fig. 13). The terminal lamellar core showed a bilateral conformation consisting of hemilamellae of presumably modified Schwann cells, surrounded by fully circular lamellae and cell nuclei (Fig. 10), while in the distal end of the corpuscles (ultraterminal region), hemilamellae were absent and the inner core cells fully encircled the axon several times (Fig. 12). The lamellae were an extension of the inner core cells and consisted of double-layered plasma membranes with scarce cytoplasm containing mitochondria, abundant pinocytotic vesicles and associated membrane invaginations, vesicular structures, likely ribosomes, and smooth endoplasmic reticulum in the bulbous enlargement of the cytoplasm near the radial clefts. The plasma membrane was covered with an extensive glycocalyx, and we noted desmosome-like junctions between adjacent lamellae. The extracellular matrix in the radial clefts and interlamellar spaces of the core mainly comprised scattered collagen fibrils, in seemingly larger clusters in the more peripheral layers. The collagen fibrils were orientated in two main directions, parallel and perpendicular to the longitudinal axis of the corpuscle, and had a diameter of about 15 nm in the extracellular matrix between the hemilamellae, and between 25 and 40 nm in the clefts and between lamellae in the ultraterminal region of the corpuscle. The corpuscles did not have an outer core or distinct lamellar capsule as do Pacinian corpuscles or a distinct connective tissue capsule as in Meissner’s corpuscles. Nonetheless, the peripheral, fully circular lamellae that contained the inner core cell nuclei, could be called a ‘capsule’ as in accordance with the morphology of Golgi-Mazzoni corpuscles (Fig. 10). Corpuscles were often found in close vicinity of nerve fibres, and there was a shared and continuous ‘perineurium’ (Fig. 10), which was morphologically not distinguishable from the peripheral layer of the corpuscles.

**Figure 14:**
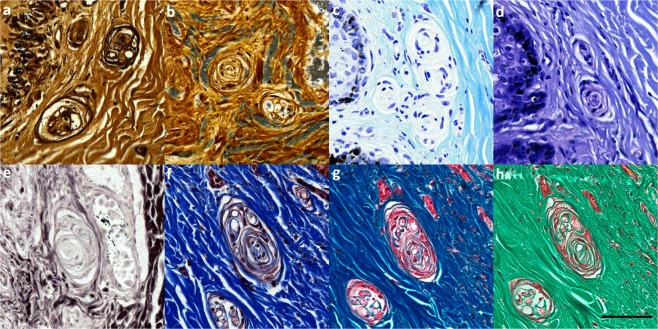
Various histochemical stains of lamellar corpuscles the subepithelial tissue of the external ear canal in striped dolphin. (**a**) Palmgren’s silver stain; (**b**) Spaethe’s silver stain; (**c,d**) Luxol Fast Blue x2 (different staining times); (**e**) Bielschowsky’s stain; (**f**,**g**) Masson’s Trichrome with Aniline x2 (different staining times); (**h**) Masson’s Trichrome Goldner. (Scale bar 50 µm).

The various histochemical staining techniques (Luxol Fast Blue/Cresyl Violet stain, Spaethe’s Silver stain modified after Richardson^14^, Palmgren’s Silver stain, Bielschowski’s Silver stain^15^, Masson’s Trichrome Goldner, and Massons Trichrome with Aniline blue) confirmed the results obtained with IHC and TEM, but did not contribute to a deeper understanding of the morphology of the lamellar corpuscles or other components of the peripheral nervous system in the ear canal (Fig. 14).

**Figure 15:**
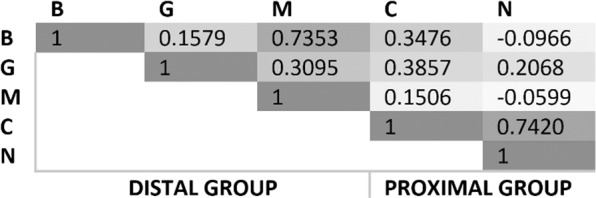
Correlations of the number of cross-sections of lamellar corpuscles among the five regions of the external ear canal (B: blubber; G: glands; M: muscle; C: cartilage; N: nervous button).

The number of corpuscle core transections in the five regions of the ear canal (B: blubber; G: glands; M: muscle; C: cartilage; N: nervous button; See also Table 1) did not present any significant interregional differences in striped dolphin. We compared the maximum values of each region among all animals, and the Spearman correlation analysis (Fig. 15) showed that region B was closely associated with M (0.7353); and C and N were strongly associated (0.7420), even though although the data was sparse as can be seen in Table 2. Based on these correlations, we merged the regions into two main groups: B + G + M (Distal group) and C + N (Proximal group), to compare the distal regions of the ear canal, with only soft tissues, to the proximal regions, which contained the cartilage. The maximum corpuscle core count between the two obtained groups was compared using the dispersion in each group, which was measured using the mean absolute deviations. These were reasonably close together (6 and 10 respectively), and a ranksum test was applied to find a difference in the median of the maximum values of Distal versus Proximal groups, which gave a p value of 0.2035 and did not allow for a rejection of the null-hypothesis of an equal median of maximum values among groups, which was already expected based on the initial group comparison. The corpuscles count for the other species are given in Table 2, but were not subjected to any analysis due to the sparsity of data.

**Table 1:** Schematic representation of soft tissues associated with the ear canal (rows), from left to right representing the course from the external ear opening to the tympanic conus.

		External ear opening	Regions for quantification*							Tympanic conus
			Blubber	Glands		Muscle	Cartilage	Nervous button		
Soft tissue	Blubber		*X*	*X*						
	Glands			*X*	*X*					
	Muscle				*X*	*X*	*X*			
	Cartilage						*X*	*X*	*X*	
	Button								*X*	

^*^Blubber: From the external ear opening to the distal end of the glands (excluding glands); Glands: containing glandular structures; Muscle: From the proximal end of the glands to the proximal end of the cartilage; Cartilage: From the distal end of the cartilage to the proximal end of the ‘nervous button’; Button: from the distal end of the nervous button to the proximal end of the ear canal.

The columns represent the regions used for quantification.

**Table 2:** Quantification of corpuscle cores: animals and manual counts.

Animal ID	Species	CC	Origin	Date of necropsy	Death	Sex	Length (cm)	Weight (kg)	Left/Right ear canal	B	G	M	C	N
12691	S. coeruleoalba	2	Livorno, Italy	19/12/2017	Natural	M	193	62	L		22	12	45	56
12703	S. coeruleoalba	2	Imperia, Spain	13/01/2018	Natural	M	133	34	L	18	25		26	
12708	S. coeruleoalba	2	Genova, Italy	21/01/2018	Natural	M	192	69	R		13		11	
109064	S. coeruleoalba	2	Calabria, Italy	13/10/2018	Natural	M	102	12	R		26		18	54
127565	S. coeruleoalba	2	Calabria, Italy	28/11/2018	Natural	M	200	na	R	14	29		19	32
N-419\16	S. coeruleoalba	2	Port de la Selva, Spain	16/11/2016	Natural	M	103	14	L	20	22		20	
N-044\17	S. coeruleoalba	2	Port Ginesta, Spain	10/02/2017	Natural	M	153	37	L	29	23		16	9
									R	16	27	14	13	
N-168\17	S. coeruleoalba	2	L’Escala, Spain	19/04/2017	Natural	M	193	79	L	24	19	17	5	15
N-488\17	S. coeruleoalba	2	Gavà, Spain	28/09/2017	Euth.	F	198	70	L	8	29		7	
N-509\17	S. coeruleoalba	2/3	Tarragona, Spain	10/10/2017	Natural	M	183	87	L	24	24	22	23	28
N-620\17	S. coeruleoalba	2	Vilanova i la Geltrú, Spain	24/07/2018	Natural	M	196	75,5	L	7	11	4	9	14
N-042\18	S. coeruleoalba	2	Viladecans, Spain	25/01/2018	Natural	M	178	62	L		43	21	13	12
N-077\18	S. coeruleoalba	2	Delta del Ebro, Spain	19/02/2018	Natural	M	220	97,5	R	11		14	20	
N-145\18	S. coeruleoalba	2	Palamós, Spain	26/03/2018	Natural	F	195	91	L				22	21
N-274\18	S. coeruleoalba	3	Delta del Ebro, Spain	24/06/2018	Natural	M	152	38	L		34	10		26
									R	22	20		24	23
N-292\18	S. coeruleoalba	2	Tarragona, Spain	06/07/2018	Natural	M	194	59,5	L	13	21	20	12	15
N-293\18	S. coeruleoalba	3	Port Ginesta, Spain	09/07/2018	Euth.	F	187	85	L		16		1	2
N-362\18	S. coeruleoalba	2	Riumar, Spain	12/09/2018	Euth.	M	181	78	L	12	9		13	18
N-169/17	D. delphis	2	L’Escala, Spain	20/04/2017	Natural	M	179	72	L	10	16		8	
									R		9	9	9	
444	T. truncatus	3	Pellestrina, Venezia, Italy	24/03/2018	Natural	M	264	260	L	33	30		40	30
									R		26		18	54
457	T. truncatus	2	Bibione, Venezia, Italy	29/01/2019	Natural	M	285	260	R	8	19		17	26
429	Z. cavirostris	2	Livorno, Italy	23/12/2017	Natural	M	503	na	L			18	12	

^*^CC: conservation code; Death: natural or euthanized after veterinary consultation; na: not available; B: blubber region; G: glandular region; M: muscle region; C: cartilage region; N: nervous button region.

### Other toothed whales

**Figure 16:**
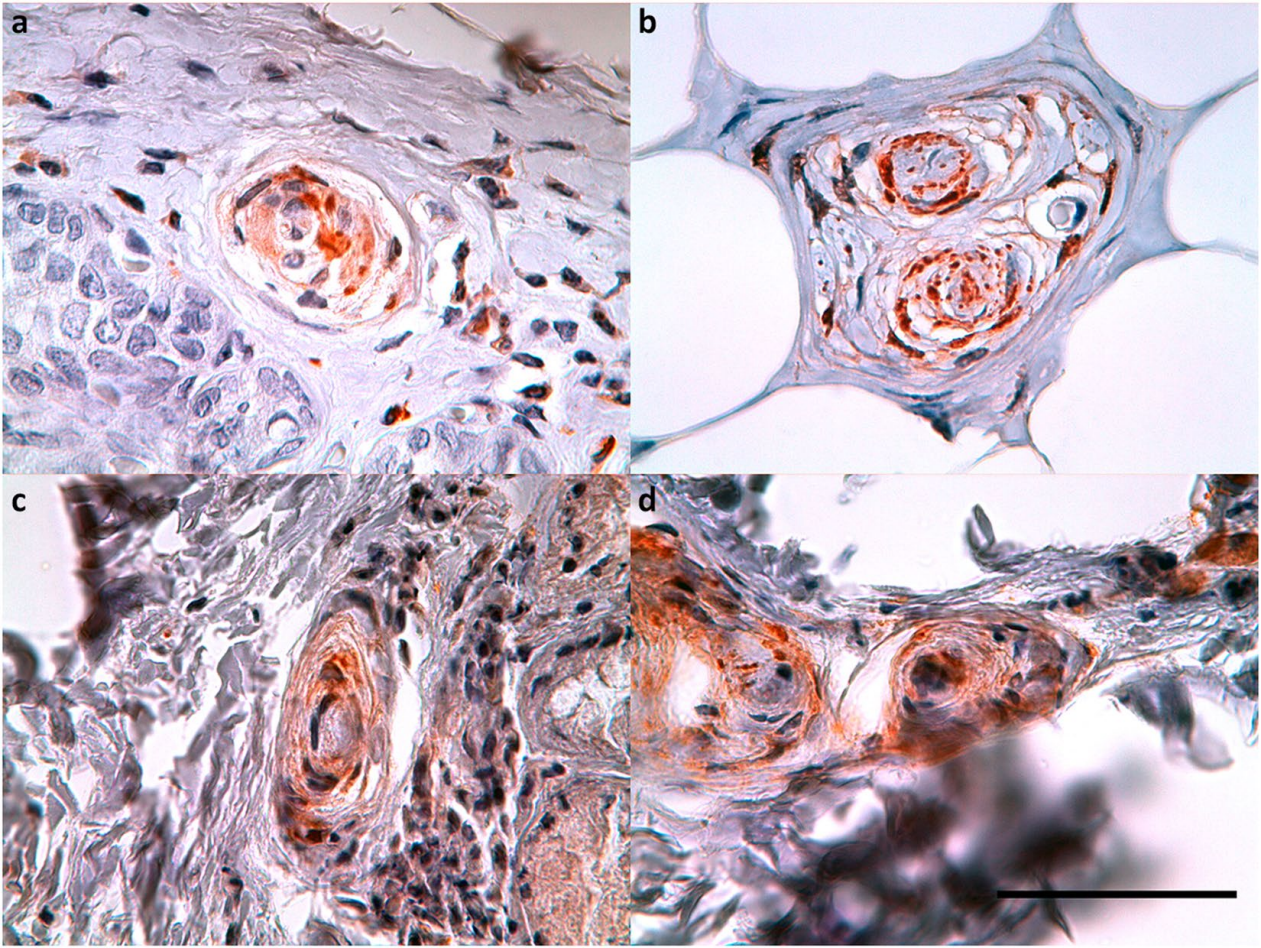
PGP 9.5 (1:500, block, no bleaching). Immunoreactive lamellar corpuscles in the vicinity of the ear canal in common dolphin (**a**), Cuvier’s beaked whale (**b**), long-finned pilot whale (**c**), and bottlenose dolphin (**d**). (Scale bar 50 µm).

The findings in all of the other odontocete species were similar to the ones in striped dolphin. They all showed the presence of small nerve fibres, lamellar corpuscles, and the same immunoreactivity for all antibodies in the subepithelial tissue throughout the ear canal (Fig. 16). The situation in the common and bottlenose dolphin was exactly the same as in the striped dolphin, with many lamellar corpuscles and small nerve fibres close to the epithelium in all sections. Also, the long-finned pilot whale and Cuvier’s beaked whale presented lamellar corpuscles and small nerves in all sections, but they were difficult to distinguish from one another because of the condition state of the tissue.

### Terrestrial cetartiodactyla

**Figure 17:**
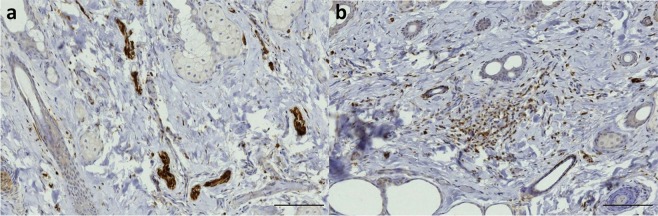
(Giraffe, anti-S100). (**a**) Immunoreactive small nerve fibres in the vicinity of sebaceous glands, and small positive reaction associated with hair follicles. (**b**) Concentration of immunoreactive cells in the dermis (Scale bars 100 µm).

**Figure 18:**
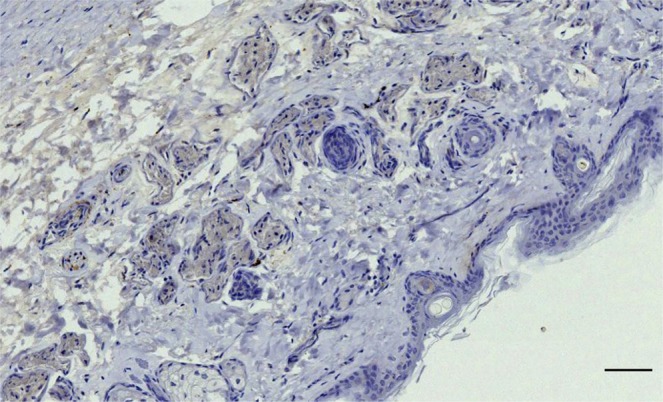
(Deer, Anti-NSE) Dermis and epidermis show no reactivity. There is unspecific staining of the glands. This image is representative for all terrestrial mammals in this study. (Scale bar 100 µm).

**Figure 19:**
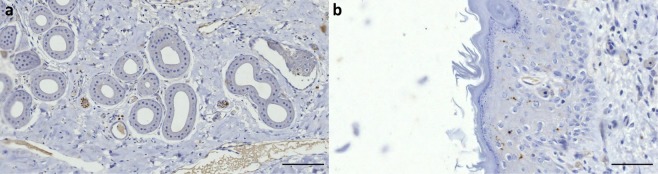
(Cow, Anti-PGP 9.5, 1:500). (**a**) Small nerve fibres in spatial association with alveolar glands. There is also unspecific staining of serum in the blood vessels (scale bar 100 µm). (**b**) intraepithelial nerve fibres (scale bar 50 µm).

The ear canal of all terrestrial Cetartiodactyla showed innervation with nerve fibres running along the canal, small nerve fibres associated with the dermal glands and hair follicles (Figs. 17 and 18), and intraepithelial free nerve endings (Fig. 19), which were the only type of SNF we could discern with certainty. All animals showed immunoreactivity for all four antibodies used, although the nerve fascicles in the deer labelled relatively weak for anti-NSE. Also, anti-S100 stained other structures such as the alveolar cells of the glands, the endothelium of vascular structures, and possibly Langerhans cells dispersed in the loose connective tissue of the dermis (Fig. 17).

## Discussion

This paper, to the best of our knowledge, is the first in describing the innervation of the ear canal in striped dolphin, bottlenose dolphin, common dolphin, long-finned whale and Cuvier’s beaked whale. Although the ear canal of these species has been previously studied, no attention was given to nervous structures^10^.

Although we noted the presence of lamellar corpuscles throughout the entire course of the canal, a histological quantification has not yet been performed. Such a presence indicates the sensitivity of this organ with a possibly graded sensory system. Although similar corpuscles have been mentioned for both whalebone and toothed whales in a variety of tissues including the skin of the belly, back, lips, eyelids and iridocorneal angle, blowhole and nasal sac system, and vibrissal crypts^8–11,16,17^, most records provide only a brief remark of their presence, with few studies giving detailed descriptions of ultrastructural features^10,16^. The present communication confirms previous assumptions on the nature of the different components that make up the corpuscles, and shows that they would function as mechanoreceptors. Our findings also show similarities with the morphology of ‘simple lamellar corpuscles’ in the skin of land mammals, except that in terrestrial mammals these only occur in association with hair^18^. These corpuscles have been differently named in the literature: Krause cylindrical corpuscle^19^, small Vater Pacini corpuscles^9^, corpuscle of Rochon-Duvigneaud^16^, laminated corpuscles^6^, simple lamellar corpuscle or Golgi-Mazzoni corpuscle (depending on the definition)^20^, encapsulated corpuscles, paciniform corpuscles, etc. We propose to use an unambiguous nomenclature for future references.

The immunoreactivity of the central axon was similar to Pacinian corpuscles in terrestrial mammals^21^. The course of the axon seemed to be straight throughout most corpuscles, although occasionally convoluted. Comparatively, the central axon of lamellar corpuscles in the eye of a beluga whale also showed a tortuous course^16^, and so does the supplying axon on entering Pacinian corpuscles in terrestrial mammals^18^. Also, the presence of accessory axons within or associated with the peripheral layer has been described in Pacinian corpuscles^18,22^, and in the capsule of tendon organs^23^, and are generally accepted to be catecholaminergic fibres that can adjust the responsiveness of the corpuscles^18,22^. However, we do not know if these are truely ‘accessory’, as the innervation patterns of the corpuscles are not yet understood. The core lamellae showed IR for anti-S100 protein, indicative of Schwann receptor cells, similar to the inner core of Pacinian corpuscles^18,24,25^. We often noted a space between lamellae and peripheral layer, similar to the acellular space between the inner core and the intermediate layer in Pacinian corpuscles^26^, although we suspect this to be an artefact associated with tissue conservation and/or processing. The peripheral layer showed unexpected IR for anti-PGP 9.5, unlike the outer core of Pacinian corpuscles^24^, and not for anti-S100, while the perineurium of nerves displayed the opposite reaction. This would indicate a difference in nature between the peripheral layer of the corpuscles and the perineurium of nerves. As such, we hypothesize that the peripheral layer could be similar to the intermediate layer of Pacinian corpuscles, which is regarded as an endoneurium with modified endoneurial fibroblasts^26^, but more investigations should be done to identify the nature of this layer.

The unanimous labelling of subepithelial nervous structures is considered to be a true positive reaction although it was often granular in form, similar to the epithelial pigmentation. This feature is specific for toothed whales, as it was absent in all terrestrial mammals. In contrast, the intraepithelial free nerve endings are a common feature among all mammals. They serve as nociceptors, mediating the sensation of pain associated with destructive mechanical stimuli, noxious chemical substances or extreme hot and cold temperatures^27^. Finally, we did not note any other types of SNF such as Merkel cells associated with the ear canal of any species, although these have been seen in the skin of cetaceans using PGP9.5^28^.

The herein investigated antibodies cross-reacted against formalin-fixed, paraffin-embedded tissues of stiped and bottlenose dolphins, reflecting the immune reaction patterns already showed in other species of terrestrial mammal. To validate antibody specificity, other than the correct use of positive and negative samples, the immunoreactivity against the proper cetacean antigen was proved by Wester Blotting analyses, as suggested by standard guidelines for veterinary laboratories^29^.

The morphology of the lamellar corpuscles as shown by transmission electron microscopy was overall consistent with the morphology of the inner core of Pacinian corpuscles, and there were also resemblances to Golgi-Mazzoni corpuscles^30^, but with significant differences, such as the likely single nerve fibril innervation and the morphology of the axon terminal with cytoplasmic processes of the inner core cells, which was without any obvious asymmetry in our results. We could identify at least two corpuscle regions: the terminal and ultraterminal region, both of which showed similarities to Pacinian corpuscles in the morphology of the axon terminal, its content, radial clefts and the hemilamellar structure in the terminal region, and the fully encircling lamellae in the ultraterminal region. The radial clefts were not described in previous electron microscopy studies of corpuscles in the iridocorneal angle^16^, and in the nasal sac system of toothed whales^10^. Also, we did not note an axon enlargement or bulb at the end of its course as mentioned for corpuscles in the ear canal of a giant beaked whale^6^ and for Pacinian corpuscles^18^, although we possibly missed it in the paucity of available tissue. The lamellar and hemilamellar structures were consistent with the morphology and size of Pacinian corpuscle inner cores, including the cellular cytoplasm, glycocalyx and desmosomal connections, down to the diameter of extracellular collagen fibres^18,31,32^. Although the corpuscles did not have a distinct outer core/capsule, there was a continuation of the peripheral layer of some corpuscles with the perineurium of nerve fibres, and the distinction between inner core lamellae and the peripheral layer was not clear, while these did show distinct reaction in the immunohistochemical study. The presence of myelin observed in the TEM images might have been affected by post-mortem degeneration, as the samples used for this study were fixed 22 hours post-mortem. With this delay of fixation, it is common to observe degeneration of the myelin sheath. To differentiate between inner core, a possible intermediate layer as in Pacinian corpuscles and an outer core/capsule, future studies could look at performing IHC with e.g. anti-collagen II, anti-collagen V, anti-S-100 and GFAP^33^. More data on the 3D morphology of the corpuscles would be needed for a better understanding of their function.

The results on the quantification of the interregional density distribution of the lamellar corpuscles showed indications that there are no interregional distribution differences in the quantity of cross-sections through lamellar corpuscle core in striped dolphin. Unfortunately, the number of specimens from other species, and their tissue condition state, did not allow for a reliable comparative analysis. We did find lamellar corpuscles in all species, including Cuvier’s beaked whale, unlike what was described for giant beaked whale where all corpuscles were concentrated in the proximal half of the ear canal^6^. Design-based stereological methods would be needed to investigate this further, looking at intraspecific and interspecific differences, and would help to understand the function and sensitivity of the ear canal in species with different physiological adaptations.

Regarding the various histochemical staining techniques, including specialized silver stains applied to formalin fixed tissues, these did not contribute to a better understanding of the morphology of the lamellar corpuscles. However, all stains were applied to opportunistically obtained formalin-fixed tissue, which is what also impeded us from applying other techniques. Future prospective studies could look at appropriate fixation protocols to comply with various histochemical techniques, especially regarding specialized silver stains, for the visualization of the peripheral nervous structures.

The presence of simple lamellar corpuscles in several delphinid species, a long-finned pilot whale and a Cuvier’s beaked whale, together with literature references for several other species, including baleen whales^6,7^, we can assume that it is a common characteristic among cetaceans. The lack of such SNF in terrestrial mammals indicates that this feature is an evolutionary adaptation of the ear canal of cetaceans to life in an aquatic environment. In comparison, there is an online mentioning of Pacinian corpuscles, Meissner corpuscles and hair follicle receptors in the ear canal of humans, associated with a reflex mechanism that would serve to expel foreign objects by means of glandular secretion and muscular contraction^34^, while we could not find morphological evidence of such SNF in the cited literature^35–37^. Moreover, a recent study that used immunohistochemistry with NF, S100, and MBP (myelin basic protein), stated that there were no SNF in the cavum conchae or cartilaginous portion of the external ear canal in humans^38^. The ear canal of all terrestrial mammals that have been studied is well-innervated^39–43^, but how it is triggered by external stimuli is not clear.

The somatosensory innervation of the ear canal in terrestrial mammals has connections to the trigeminal (V), facial (VII), glossopharyngeal (IX), vagal (X) cranial nerve ganglia, and also the C2–C4 dorsal root ganglia^40–44^. There are two peripheral nerves responsible for relaying afferent impulses from the ear canal to the ganglions of the cranial nerves, namely the auriculotemporal nerve (<V_3_) and the auricular branch of the vagal nerve. These nerves supply specific locations of the ear canal walls, but with an overlapping distribution^44^. In dolphins, the only major nerve that has been described innervating the region of the external ear canal is the auriculotemporal nerve (<V_3_)^45^. The trigeminal nerve in cetaceans contains an exceptionally high number of nerve fibres, and is responsible for the sensory innervation of several sensitive facial structures including the eyes, lips, and blowhole, but also the mandibles and nasal sac system^12,45–47^. It also has motor functions, and has been proposed to be associated with the regulation of the middle ear volume through the blood supply of the corpus spongiosum^48^, and innervates the entire facial musculature^11^. The external ear canal with its lamellar corpuscles might contribute to the size of the trigeminal nerve in cetaceans, as it is densely innervated with novel sensory nerve formations.

While the lamellar corpuscles have been described in both toothed and whalebone whales, in a variety of soft tissues, their exact function is still unknown. They have been described/found in the skin surrounding the blowhole and the authors associated them with the detection of ‘pressure changes’ when passing from water to air and vice versa^8^. They are also present in the iridocorneal angle of various odontocete species, where their function could be associated with mechanical deformations, such as those caused by internal pressure differences, vascular volume change, and the author even speculated on a thermosensory function^16^. In mysticetes, corpuscles have been described in the skin of the fin whale, most prominently in the lips and eyelids^9^, which are considered relatively sensitive areas in dolphins^49^. Corpuscles have also been demonstrated in the nasal sac system of striped dolphin and harbour porpoise^11,12^, and in the ear canal of several odontocete and mysticete species^6,7^. To get a better understanding of the biomechanical properties of the corpuscles, further immunohistochemical characterization could be achieved with the use of antibodies for neuronal and neurotransmitter-related molecules, possibly using confocal microscopy as this would also provide information on the 3-dimensional structure. Such information could be used for modelling and to mimic the response of these corpuscles to various stimuli.

In this preliminary hypothesis we do not exclude that the receptors could be sensitive to multiple types of stimuli, but the commonly accepted one, which is also consistent with our results, is that of mechanical deformation. Among the main stimuli that can trigger a deformation of the soft tissues of the ear canal in cetaceans, we consider (a) changes in ambient hydrostatic pressure, (b) vibratory or pulsed stimuli coming from the natural environment and/or from other animals such as conspecifics, and unnatural sources such as anthropogenic noise (this would allow them to hear or feel the environment, depending on the perspective), and finally, (c) touch, although less likely because an associated behaviour has never been reported. For the detection of ambient pressure, the suited type of mechanoreceptors would have a slow adaptation rate, while for transient deformation such as vibration, it would be rapidly adapting. The lamellar corpuscles described here, show an immunohistochemical and ultrastructural resemblance to the inner core of a Pacinian corpuscle, a rapidly adapting, low-threshold mechanoreceptor^18,21,31,32^. However, an artificial removal of the outer core of the Pacinian corpuscle lowers its adaptation rate^50^. This could allow the corpuscles described here to be stimulated by steady pressure^27^. Also, the absence of the outer lamellar core would omit the amplitude and frequency filtering mechanism it accommodates^18,51^, leading to a corpuscle that can be stimulated by smaller deformations over a wider frequency range. Therefore, although we cannot make definite conclusions on the adaptation rate of the lamellar corpuscles described here, we hypothesize they could be appropriate for the sensation of pressure, and the associated perception of depth. Such information would be essential to accommodate physiological responses associated with diving, through general body responses, after processing in the central nervous system, or through arched reflexes in structures that are connected to the same neural ganglia. One of the most striking adaptations of toothed whales is the ability to cope with great pressure differences through vascular perfusion and the presence of extensive arterial and venous plexuses and retia mirabilia^47,52,53^. Connected to the same neural ganglia, are the middle ear and its corpus spongiosum, and the mandibular fat bodies and accessory sinus complex with extensive venous plexuses and prominent arterial supply^54^, which are all novel evolutionary structures subjected to large pressure differences and showing considerable physiological flexibility, and are essential for a correct functioning of the hearing apparatus at depth. While there are indications of pressure-related redistributions of blood, at least in the middle ear and pterygoid sinuses, it is not yet known how those might be regulated. For comparison, there are indications of active vasodilation of the vascular lacunae in the dolphin trachea with nitric oxide as chemical messenger^55^, possibly coordinated through a centrally stimulated parasympathetic innervation^27^. It would be interesting to study how the blood supply in the middle ear and associated tissues is controlled.

The working hypothesis, supported by our results and literature findings, is that the ear canal has acquired a new sensory role and would be able to detect the (changes in) ambient pressure, while the topographical distribution of the lamellar corpuscles, and the intricate association with complex configuration of the lumen and the surrounding soft tissues, could allow for a graded sensation.

## Conclusion

In this study, we have clarified the structural nature of simple lamellar corpuscles associated with the external ear canal in toothed whales and given insight into the complex innervation of the canal. It shows that the presence of lamellar corpuscles is a feature that is unique to cetaceans, not present in terrestrial Cetartiodactyla, which indicates that, through their return to the aquatic environment, the external ear canal has acquired a unique sensory function. While the function is still a conundrum, we propose a hypothesis based on morphological data in a variety of toothed whales and propose it to be an organ capable of detecting ambient pressure through which information can be relayed to physiological reflex responses essential for underwater hearing. More information on the three-dimensional characteristics of the corpuscles, and the conformation of the ear canal soft and cartilaginous tissues would be required to gain insight in the physiology, sensitivity, and function of the external ear canal. Although we cannot draw definite conclusions yet, this paper provides essential data for a better understanding of the definite functionality of the odontocete ear canal.

## Materials and Methods

### Animals

For the present study, we used several specimens of striped dolphin, two bottlenose dolphin, and single specimens of common dolphin, long-finned pilot whale, and Cuvier’s beaked whale, and also several terrestrial Cetartiodactyla (i.e. cow, roe deer and northern giraffe). A detailed list of the examined animals is reported in Tables 2 and 3.

**Table 3:** Animals for IHC.

Animal ID	Species	CC/CS	Origin	Date of necropsy	Death	Sex	Length	Weight	PGP9.5	NF	S100	NSE
12691	*S. coeruleoalba*	2	Livorno, Italy	19/12/2017	Natural	M	193 cm	62 kg	x	x	x	x
12708	*S. coeruleoalba*	2	Genova, Italy	21/01/2018	Natural	M	192 cm	69 kg	x	x	x	
*N-293\18*	*S. coeruleoalba*	3	Port Ginesta, Spain	09/07/2018	Euthan	F	187 cm	85 kg	x	x		x
*N-274\18*	*S. coeruleoalba*	3	Delta del Ebro, Spain	24/06/2018	Natural	M	152 cm	38 kg		x	x	x
*N-488\17*	*S. coeruleoalba*	2	Gavà, Spain	28/09/2017	Euthan	F	198 cm	70 kg	x		x	x
444	*Tursiops truncatus*	3	Pallestrina, Italy	24/03/2018	Natural	M	264 cm	260 kg	x			
*N-169\17*	*Delphinus delphis*	2	L’Escala, Spain	20/04/2017	Natural	M	179 cm	72 kg	x			
429	*Ziphius cavirostris*	2	Livorno, Italy	23/12/2017	Natural	M	503 cm	na	x			
441	*Globicephalus melas*	4	Aglientu, Sardinia, Italy	20/03/2018	Natural	M	525 cm	na	x			
*AE246*	*Bos taurus*	Good	Venice, Italy	12/12/2018	Natural	F	na	100 kg	x	x	x	x
*CP11*	*Capreolus capreolus*	Mod/Adv	Prato, Italy	30/11/2017	Natural	M	na	14 kg	x	x	x	x
*AB959*	*Giraffa camelopardalis*	Good	Verona, Italy	01/03/2018	Natural	M	na	459 kg	x	x	x	x

^*^CC/CS: conservation code or status; Death: natural or euthanized after veterinary consultation; na: not available; x: antibody applied.

### Tissue sampling and processing

The external ear canals of all animals were obtained during routine necropsy. The odontocete samples were taken according to the following protocol: First, the external ear opening was located and soft tissues were dissected in a rectangle of about 1 cm^2^ around the opening down to the level of the osseous cove that is delineated by the mandible rostrally, the squamosal dorsally, and the exoccipital bone dorsocaudally. Next, the soft tissues that hold the TP-complex in place were cut and the combined tissues of the ear canal, surrounding soft tissues and TP-complex were dissected and extracted. The entire structure was fixed in 10% neutral-buffered formalin for 24 hours after which the fixative was refreshed. The time from fixation to tissue processing ranged from several days to 18 months. The TP-complex, together with the far-most medial end of the ear canal was separated and decalcified using a commercial decalcifier (Biodec R, Bio-Optica®). For the terrestrial mammals, we dissected the cartilaginous portion of the external ear canal, which we fixated in the same manner as the odontocete samples. Next, slabs with a diameter of about 3–4 mm were dissected, transverse to the local orientation of the ear canal lumen, embedded in paraffin, sectioned to a thickness of 4 µm, and mounted on polarized glass slides. Sections for staining with hematoxylin-eosin were obtained from all slabs and dried overnight at 70 °C, followed by automated staining using a Leica Autostainer XL (Leica Biosystems Nussloch GmbH). The slides were coverslipped using a mixture of Eukitt^®^ (ORSAtec GmbH) and xylene.

### Immunohistochemistry

Slides for immunohistochemistry were dried at room temperature and incubated at 37 °C for 30 min before the start of the semi-automatic staining procedures using Benchmark® GX and Ventana software, using Polyclonal anti-bovine S-100 (Dako, Z0311), Monoclonal anti-human Neurofilament M Protein (NF)(Dako, M0762), Monoclonal anti-human Neuron-Specific Enolase (NSE)(Dako, M0873), and Polyclonal anti-bovine PGP 9.5 (Dako, Z5116) as primary antibodies in several dilutions with and without blocking agent (See Supplementary Material). The sections for anti-PGP 9.5 were stained with a protocol modified from^56^, which, in some odontocete cases, was preceded by a melanin bleaching procedure (modified from^57^). Antibodies were diluted with Ventana^®^ Antibody Diluent with or without casein blocking buffer. After staining, all slides were washed with standard dishwashing soap and rinsed with tap water for several repetitions during 10–15 min. Next, the slides were dehydrated in increasing concentrations of alcohol, coverslipped and dried overnight at room temperature inside the extraction hood. The specificity of the immunohistochemical reaction was checked using a) negative control (internal and external control with epithelium, muscle, fat, and connective tissue), and b) white control sections (primary antibody absent) (See Supplementary Material). All slides were examined with an Olympus BX41 microscope (Olympus Italia S.r.l., Milan, Italy) at up to x600 magnification, and scanned with D-sight scanning microscope at x400 magnification (A. Menarini Diagnostics, S.r.l., Florence, Italy) and uploaded to a server (Telepathology, Visia Imaging S.r.l., San Giovanni Valdarno (AR), Italy), and pictures were taken either as screenshots from the server or with a Leica DMD108 Microscope (Leica Microsystems CMS GmbH, Milan, Italy). Images were edited using Fiji software (ImageJ 1.52i) for adding the scale bar, for appropriate brightness and contrast when insufficient, and montages were made using the Magic Montage plugin. For the classification of the SNF, we compared morphology with literature (See Malinovský, 1996, for a review).

### Histochemical stains

Slides for histochemical stains were obtained in the same manner as for standard haematoxylin-eosin. The techniques included: Luxol Fast Blue / Cresyl Violet Stain, Spaethe’s Silver stain modified after Richardson^14^, Palmgren’s silver stain, Bielschowski’s silver stain^15^, Masson’s Trichrome Goldner, and Massons Trichrome with Aniline blue.

### Protein extraction and western blotting analysis

For protein extraction, 150 μg of frozen brain tissue from bottlenose dolphin, striped dolphin and bovine (closest phylogenetic species in which cross-reactivity tissue controls were possible) were homogenized using Potter glass (Vetrotecnica, Italia) in 5 ml of buffer A (10 mM Tris-Base, 150 mM NaCl, 5 mM EDTA, pH 7.2 and cocktail inhibitor - Sigma, Milan, Italy) and centrifuged at 10000 g for 30 minutes. The supernatant was then centrifuged at 125000 g for 1 hour (Optima L-90K, Beckman, Italy) and the pellet proteins were dissolved in 0.5 ml of buffer B (10 mM Tris, 150 mM pH 7.2 NaCl). Total protein concentration was calculated using the Pierce BCA Protein Assay Kit (ThermoFisher Scientific). In order to evaluate PGP 9.5, NF, NSE and S-100 antibody specificity, a Western Blot analysis has been performed according to the following protocol. Twenty-five micrograms of the extracted proteins were denatured at 70 °C for 10 minutes. Proteins were resolved using NuPAGE 4–12% Bis-Tris gel (ThermoFisher Scientific) and transferred to the nitrocellulose membrane. Nonspecific binding sites were blocked for 1 hour in 5% nonfat dry milk in TBS-T (TBS containing 0.05% Tween-20) at room temperature. Blot was incubated at 4 °C overnight with antibodies against PGP, NF, NSE and S-100, respectively (Dako). Then, the membrane was incubated for 1 hour at room temperature with anti-rabbit peroxidase-conjugate secondary antibody (ThermoFisher Scientific, #32260) for PGP and S-110 and with anti-mouse peroxidase-conjugate secondary antibody for NF and NSE (ThermoFisher Scientific, #32230). Reactive bands were visualized with a chemiluminescent detection kit (SuperSignal West Pico Chemiluminescent Substrate, ThermoFisher Scientific) using the iBright instrument (ThermoFisher Scientific).

### Transmission electron microscopy

The ear canals of a striped dolphin stranded in Delta de l’Ebre, Paya del Serrallo, Sant Jaume d’Enveja, Tarragona, Spain (23/06/2018, N-274/18, male, condition code 3, 38 kg, 152 cm length) were dissected during routine necropsy at the Veterinary Faculty of the Autonomous University of Barcelona (UAB, Bellaterra) about 22 h after the animal died on the beach without human intervention. A subsample was taken from a tissue zone medial to the blubber layer and fixed for 48 h at 4 °C in a solution of 2.5% glutaraldehyde in 0.1 M cacodylate buffer solution, subsequently post-fixed with 1% osmium tetroxide, and dehydrated with increasing concentrations of acetone in water. Semi-thin sections (1 mm) were obtained with a glass knife, then stained with methylene blue, covered with Durcupan, and observed by light microscopy. Ultrathin slides, about 100 nm thick, were cut with an Ultracut E ultramicrotome (Reichert-Jung). Sections were double-stained with uranyl acetate and lead citrate and observed with a Jeol JEM-1010 Electron Microscope (JEOL Corp. Ltd., Tokyo, Japan) at 80 kV. Images were obtained with a Bioscan camera model 792 (Gatan) at the University of Barcelona (Unitat de Microscòpia Electrònica (TEM/SEM), Centres Científics i Tecnològics).

### Corpuscle core quantification

We did a relative quantification of the number of transections through corpuscle cores over the course of the external ear canal in striped dolphin. Histological slides were obtained as described above. In each slide, the corpuscle cores were manually counted at a magnification of 200×, in all fields of view of the subepithelial tissue around the ear canal, always including the basal layer of the epithelium within the field of view. Each corpuscle core was labelled as positive only when a central axon and surrounding lamellar structure were recognized, independent of the occurrence as single corpuscles or as multiple corpuscles that were adjacent to each other or embedded within the same peripheral layer. The tissue sections were labelled as belonging to one of five distinguishable regions along the course of the ear canal (see Table 1 for a schematic representation of the presence of the different types of soft tissues over the course of the canal). Because there was a limited amount of good quality samples, we included all possible animals and sections that allowed for a reliable counting of the corpuscles. We assumed that the number of corpuscle cores would not be affected by age, length, sex, left versus right, and that it would not change within a region. We took the maximum value of all slabs in each of the five regions for further analysis, as it was considered to be most representative of the true number of corpuscles and less influenced by artefacts.

## Supplementary information

Supplementary information.
