# Supplementary material for: Morphological Evidence for the Sensitivity of the Ear Canal of Odontocetes as shown by Immunohistochemistry and Transmission Electron Microscopy
Source: Sci Rep. 2020 Mar 6;10:4191. doi: 10.1038/s41598-020-61170-4 (PMC7060263; doi:10.1038/s41598-020-61170-4)
Supplement: Supplementary file 1 — Supplementary information. [file 41598_2020_61170_MOESM1_ESM.pdf]

SUPPLEMENTARY MATERIAL

Morphological Evidence for the Sensitivity of the Ear Canal of Odontocetes as shown by Immunohistochemistry and Transmission Electron Microscopy

Steffen De Vreese[1,2*], Michel André[2], Bruno Cozzi[1], Cinzia Centelleghe[1], Mike van der Schaar[2], and Sandro Mazzariol[1]

[1] Department of Comparative Biomedicine and Food Science, University of Padova, 35020 Legnaro (Padova), Italy
[2] Laboratory of Applied Bioacoustics, Technical University of Catalunya, BarcelonaTech, 08800 Vilanova i la Geltrú (Barcelona), Spain
[*]steffen.devreese@studenti.unipd.it / steffen.devreese@lab.upc.edu

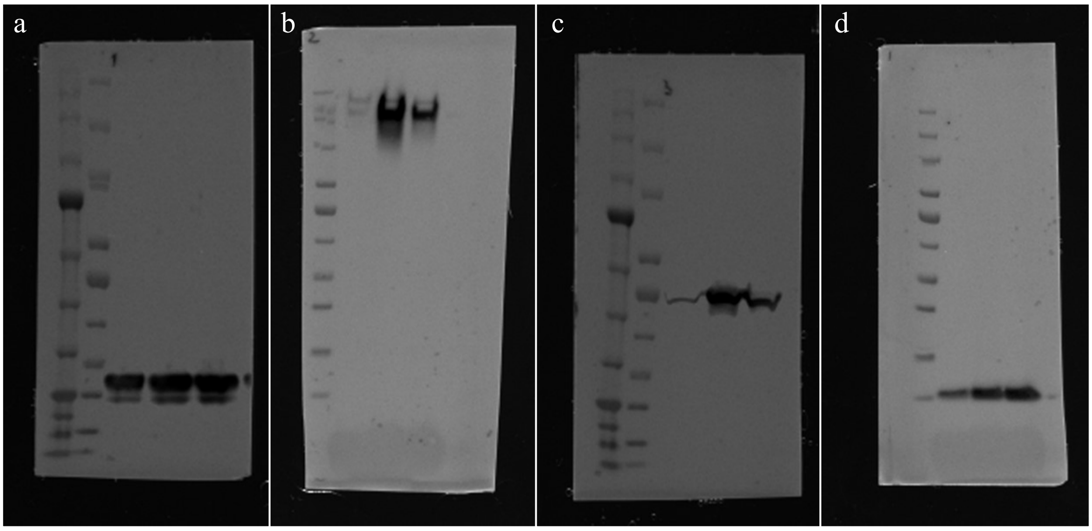

*Figure 1. Western blot analyses – Original images of full-length gels. a) Polyclonal rabbit anti-bovine PGP antibody (code Z5116, Dako) at a dilution of 1:500.; b) Monoclonal mouse anti-human NF (Clone 2F11) antibody (code M0762, Dako) at a dilution of 1:500; c) Monoclonal mouse anti-human NSE antibody (Clone BBS/NC/VI-H14) (code M0873, Dako) at a dilution of 1:500; d) Polyclonal rabbit anti-bovine S-100 (code Z0311, Dako) at a dilution of 1:1000. *: Bt: Bos taurus, Sc: Stenella coeruleoalba, Tt: Tursiops truncatus; kDa: kilodalton*

TABLE 1. Primary Antibodies

Antibody	Origin	Code	Validation	Cross-reactivity	Ag sequence conservation**	Dilution + agent
Polyclonal Anti-Bovine S-100	Rabbit	Z0311	Human*	bovine, cat, horse, mouse, swine, rat*	Sperm whale - human 100%<br>Sperm whale - bovine 97%<br>Beluga whale - bovine 98%	1:1000 (with and without block)<br>1:2000 (no block)
Monoclonal Anti-Human NF (Clone 2F11) Interacts with the NF-M subunit [1]	Mouse	M0762	Human*	bovine, swine, rabbit, mouse, horse, dog, cat*, opossum[2]	Bovine - human >99%<br>Swine - human 97%	1:100 (with and without block)
Monoclonal Anti-Human NSE (γγ-isoenzyme)	Mouse	M0873	Human*, guinea pig*, dog[3, 4]	bovine[4], dog[3,4], goat[4], pig[4], rabbit[4], rat[4], fish (*Oryzias latipes*)[5], domestic ferret (*Mustela putorius furo*)[5], cat[6], African green monkey (*Macaca fascicularis*)[7], mouse[8], Tasmanian devil (*Sarcophilus harrisii*)[9]	Goat - human >98%<br>Rat - human >98%	1:250 (with and without block)
Polyclonal Anti-Bovine PGP 9.5 (UCT-H1)	Rabbit	Z5116	Human, mouse*	None	Bovine - human >97%<br>Bovine - rat/mouse >97%	1:500 (with/without block/melanin bleaching)<br>1:2000 (with/without block, with melanin bleaching)

NF: Neurofilament Protein
NSE: Neuron-Specific Enolase
PGP: Protein Gene Product
All antibodies were supplied by DakoCytomation (Dako Denmark A/S, Denmark)
** Manufacturer data sheet*
***Calculated with use of the database of the United States' National Center for Biotechnology Information (https://www.ncbi.nlm.nih.gov/).*

TABLE 2. IHC: positive and negative controls

	CTRL+	*CTRL-*
PGP 9.5	Bovine cerebrum; Bovine sciatic nerve	Bovine skin and muscle (epithelium, connective tissue, adipose tissue, striated muscle, blood vessels)
S100	Bovine cerebrum; Bovine sciatic nerve; Canine mammary gland (myoepithelial cells)	Bovine skin and muscle (epithelium, connective tissue, adipose tissue, striated muscle, blood vessels)
NF	Cat thalamus	Cat skin with epithelium, connective tissue, striated muscle, and fat tissue
NSE	Cat thalamus	Cat skin with epithelium, connective tissue, striated muscle, and fat tissue

References

1. Klück, P. *et al.* Hirschsprung's disease studied with monoclonal antineurofilament antibodies on tissue sections. *The Lancet* **323**, 652–654 (1984).
2. Breckenridge, L. J., Sommer, I. U. & Blackshaw, S. E. Developmentally regulated markers in the postnatal cervical spinal cord of the opossum Monodelphis domestica. *Dev. Brain Res.* **103**, 47–57 (1997).
3. Fyfe, J. C. *et al.* Inherited neuroaxonal dystrophy in dogs causing lethal, fetal-onset motor system dysfunction and cerebellar hypoplasia. *J. Comp. Neurol.* **518**, 3771–3784 (2010).
4. Aoki, T., Tanaka, T. & Watabe, H. Purification and characterization of gamma-enolase from various mammals. *Chem. Pharm. Bull. (Tokyo)* **40**, 1236–1239 (1992).
5. Uemura, N. *et al.* Viable Neuronopathic Gaucher Disease Model in Medaka (Oryzias latipes) Displays Axonal Accumulation of Alpha-Synuclein. *PLOS Genet.* **11**, e1005065 (2015).
6. van Sprundel, R. G. H. M. *et al.* Classification of primary hepatic tumours in the cat. *Vet. J.* **202**, 255–266 (2014).
7. Cabo, R. *et al.* Brain-Derived Neurotrofic Factor and its Receptor TrkB are Present, but Segregated, Within Mature Cutaneous Pacinian Corpuscles of Macaca fascicularis. *Anat. Rec.* **298**, 624–629 (2015).
8. Kestell, G. R., Anderson, R. L., Clarke, J. N., Haberberger, R. V. & Gibbins, I. L. Primary afferent neurons containing calcitonin gene-related peptide but not substance P in forepaw skin, dorsal root ganglia, and spinal cord of mice. *J. Comp. Neurol.* **523**, 2555–2569 (2015).
9. Stammnitz, M. R. *et al.* The Origins and Vulnerabilities of Two Transmissible Cancers in Tasmanian Devils. *Cancer Cell* **33**, 607-619.e15 (2018).
10. Yui, T., Ohmachi, T., Matsuda, K., Okamoto, M. & Taniyama, H. Histochemical and immunohistochemical characterization of chordoma in ferrets. *J. Vet. Med. Sci.* **77**, 467–473 (2015).
